# Nocturnal activities and host preferences of *Phlebotomus orientalis* in extra-domestic habitats of Kafta-Humera lowlands, Kala-azar endemic, Northwest Ethiopia

**DOI:** 10.1186/s13071-014-0594-3

**Published:** 2014-12-17

**Authors:** Wossenseged Lemma, Habte Tekie, Ibrahim Abassi, Meshesha Balkew, Teshome Gebre-Michael, Alon Warburg, Asrat Hailu

**Affiliations:** Department of Parasitology, School of Biomedical & Laboratory Sciences, College of Medicine and Health Sciences, University of Gondar, Gondar, Ethiopia; Department of Zoological Sciences, College of Natural Science, Addis Ababa University, Addis Ababa, Ethiopia; Department of Microbiology & Molecular Genetics, The Institute for Medical Research Israel-Canada, Jerusalem, Israel; The Kuvin Centre for the Study of Infectious & Tropical Diseases, The Hebrew University – Hadassah Medical School, The Hebrew University of Jerusalem, Jerusalem, 91120 Israel; Aklilu Lemma Institute of Pathobiology, Addis Ababa University, Addis Ababa, Ethiopia; Department of Microbiology, Immunology & Parasitology, Faculty of Medicine, Addis Ababa University, Addis Ababa, Ethiopia

**Keywords:** Nocturnal activities, Host preferences, *Phlebotomusm orientalis*, Extra-domestic habitats, Kala-azar, Kafta–Humera lowlands, Northwest Ethiopia

## Abstract

**Background:**

*Phlebotomus orientalis* feeds on a variety of wild and domestic animals and transmits *Leishmania donovani* from hitherto unknown reservoir hosts to humans in extra-domestic habitats in the Metema - Humera lowlands. The aim of this study was to determine the nocturnal activities of *P. orientalis* and its preferred blood meal hosts.

**Methods:**

Collections of *Phlebotomus orientalis* were made by using CDC light traps to determine the density as *P. orientalis*/hour CDC trap and preference to rodents by using Turner’s traps in agricultural fields, animal shelters and thickets of *Acacia seyal* in Baeker site-1 and Gelanzeraf site-2. The blood meal sources were detected by Reverse Line Blot (RLB) of cytochrome *b* polymerase chain reaction (PCR) amplification in August, 2012 from collections of sand flies in thickets of *A. seyal* (March 2011) and dense mixed forest (July 2011) in Baeker site 1. RLB PCR involved first amplification of animal specific sequences of cytochrome *b* using PCR techniques. Then the amplified sequence was hybridized with 11 species-specific probes for domestic animals adsorbed on nitrocellulose membrane for calorimetric color detection.

**Results:**

A total of 6,083 *P. orientalis* (2,702 males and 3,381 females) were collected at hourly intervals using 22 CDC traps from January to May 2013. The peak activities of *P. orientalis* were at 1.00 a.m (134.0 ± 7.21) near animal shelters, 3.00 a.m (66.33 ± 46.40) in agricultural fields and 21:00 pm (40.6 ± 30.06) in thickets of *A. seyal*. This species was not attracted to the different species of rodents in trials carried out in March and April 2013. RLB PCR identified 7 human (28%), 9 mixed (human and cattle) (36%) and 2 cattle (8%) blood meals while 7 were unknown (28%).

**Conclusion:**

Female *P. orientalis* can bite humans in extra-domestic habitats of Kafta-Humera lowlands at any hour of the night with peak biting after midnight.

## Background

Of the total half a million kala-azar (Visceral Leishmaniasis; VL) cases in the world, 90% occur in Bangladish, India, Nepal, Sudan, Ethiopia and Brazil [1]. In Sudan and northwest Ethiopia, kala-azar is transmitted by *P. orientalis* [2-4] and it claims the lives of thousands of people [5-7]. *Phlebotomus orientalis* is found to exhibit anthropophilic behavior and it transmits VL from man to man and/or to dog and vice versa [8,9]. The dog was considered as an intermediate host between a possible sylvatic cycle and the anthroponotic cycle [9]. The large numbers of patients with post kala-azar dermal leishmaniasis (PKDL) in heavily affected villages in Sudan might also indicate a possible human reservoir and anthroponotic transmission [10]. *Phlebotomus orientalis* was also reported to be exo-phagic/exophilic (zoonotic VL) [9-13]. Zoonotic transmission of VL in east Africa was also suggested based on evidence of VL outbreaks among people that camped in uninhabited areas of eastern and former southern Sudan in addition to isolation of *L. donovani* from wild animals in the uninhabited Dinder National Park [14-16]. Experimental study on host preferences of *P. orientalis* and the isolation of *L. donovani* in east Africa have indicated that *Canis familiaris* (dogs), *Herpeistes ichneumon* (mongoose), *Genetta genetta* (genet cat), *Acomys* spp. and *Arvicanthis* spp. as preferred hosts for source of blood meals [2,13,17,18]. The result of *P. orientalis* from human bait also showed humans to be preferred hosts [4,19-21]. Attraction of *P. orientalis* towards cattle, sheep, goat, mule, donkey and horse has not been defined in east Africa as a whole, even though high *Leishmania* sero-prevalence rate for these domestic animals has been reported in eastern Sudan [22] and northwest Ethiopia [23].

In the Metema-Humera lowlands, seasonal labour migrants who stay in agricultural fields for weeding and harvest of sesame, sorghum and cotton are exposed to Zoonotic kala-azar infections from June to October [24]. In these areas, the nocturnal activities (dieal periodicity) and the peak biting hours of *P. orientalis* were not studied. Adequate knowledge on these aspects helps to reduce kala-azar incidence by avoiding contact with sand flies at that particular period. Information on the host preferences of this vector could also give important clues in identification of reservoir hosts of kala-azar. Thus the aim of this study was to elucidate the nocturnal activities and host preferences of *P. orientalis* in Kafta – Humera lowlands.

## Methods

### Study area

Kafta-Humera, Welkait and Tsegede are three districts in the Western Zone of Tigray region and the vast land at an altitude of around 600 m above sea level, which is endemic to kala-azar. Humera town (14°17’N, 036°39’E; altitude 637 m) is the capital of Kafta-Humera district and it is the sit for the administrative centers of the district and the Zone. It is bordered to Eritrea in the north and Sudan in the west. It is located 180 km west of Shirao town (Tahatay Adiyabo district of Tigray region) and 253 km northwest of Gondar town (Amhara region). Kafta-Humera district has a total population of 92,167(47,909 men and 44,258 women) and covers an area of 4, 542.33 square km [25]. In the district small towns such as Rawyan (14°17′ N, 36°37′E), May Kadra (14°08′ N, 36°34′ E), Baeker (14°00′ N; 36°61′E) and Adebay (14°17′N, 36°38′E) are surrounded by uniform agricultural fields that are occasionally interrupted by thickets of *Acacia seyal* in depressions. All the dense mixed forests were converted into agricultural fields except in the Kafta Shiraro Park and some rocky outcrops. The part of the dense mixed forest in Baeker is used for grazing of domestic animals especially during the rainy season when all arable land is covered with crops. The common trees and shrubs in Kafta-Humera areas are *A. seyal*, *A. mellifera*, *Balanites aegyptiaca*, *Terminalia* spp., *Boswellia papyrifera*, *Ficus sycomorus*, *Sclerocarya birrea*, *Zizypus* spp., *Dalbergia melanoxylon*, *Boscia angustifolia*, *Sterculia africana*, *Adansonia digitata*, *Dichrostachys cineria* and *Syzgium guineese*.

**Figure 1:**
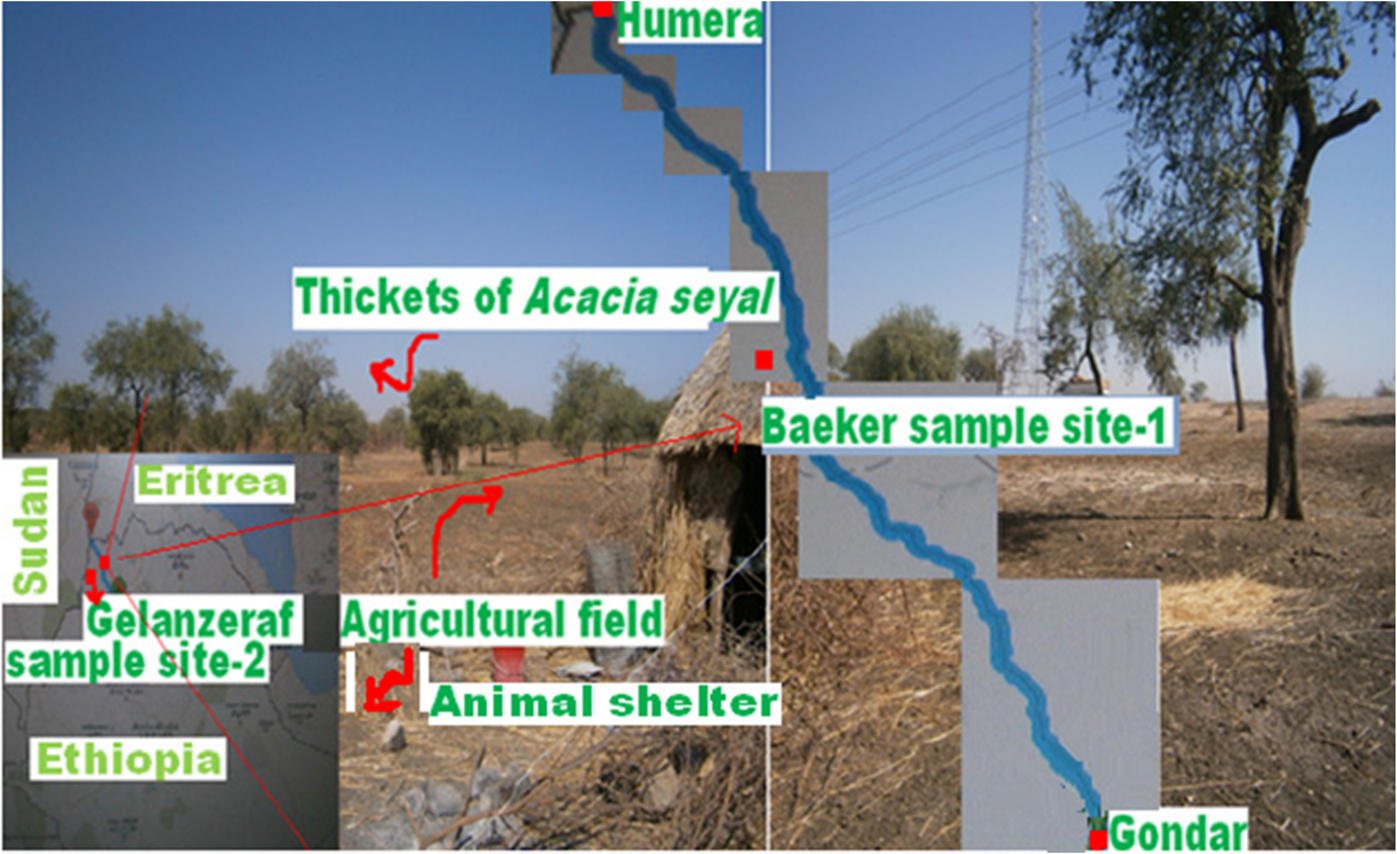
**Agricultural field, animal shelter and thickets of** ***Acacia seyal*** **sampling points in Baeker site 1, where collections of sand flies for the studies of nocturnal activities and host preference towards rodents were conducted from January to May 2013.**

The study was conducted in two selected extra-domestic sites. These were Baeker town (Site 1) and Gelanzeraf (Site 2). The former is a small town located 40 km from Humera town on the asphalt road leading to Gondar. Baeker extra-domestic sand fly sampling Site 1 (14°01’N, 36°59’E, 651 m) is located 33 km from Humera town close the main road between Humera and Gondar towns. Gelanzeraf is a locality southwest of Humera, near the Sudan boarder. Gelanzeraf extra-domestic sampling Site 2 (13°59’N, 036°31’E) is located at 38 km from Humera town near the new asphalt road leading to Sudan, at about 20 km from the Sudan border (Figure 1). In both sites, there were closely placed huts (tukuls) in the camps on the agricultural fields. Domestic animal shelters are located at about 200 m in Site 1 and 250 m in Site 2 from the huts. *Acacia seyal* in Site 1 is located about 2 km from the huts compared with 4 km in Site 2. In Site 1, the two vacant huts were giving service only during agricultural rainy season. The 4 guards near telecommunication bare (tower) and 4 animal keepers near the animal shelters were the only persons near the huts in Site 1 in addition to 3 individuals working on irrigation of vegetables, using ground water in a gorge found slightly far away from the huts. One individual, of those working in the irrigation, left the area following kala-azar infection in September, 2011, after completing treatment in Humera Kahsaye Abera hospital. In Site 2, the camp has 3 huts and a big store with 5 individuals who take care of the area and the animals.

Goats and sheep are browsing and grazing in agricultural fields and sparse thickets of *A. seyal* including the dense mixed forest (Part of Kafta-Shirao park in Baeker at about 7 km from Site 1) during the day and shelter near the camps at night unlike cattle which grazed at night during dry season (October - June). In agricultural rainy season, however, animals are guarded in the dense mixed forest and rocky uncultivated areas. Similar points on agricultural fields, animal shelter and sparse thickets of *A. seyal* were selected for the repeated sand fly collections during studies of nocturnal activities and host preference towards rodents. The sampling points on agricultural fields and animal shelter were separated by about 400 m with the huts located between them in both Site 1 and 2. The sampling point for animal shelter was about 2 m from where the goats and sheep sheltered in both sites.

### Habitats

The vegetations in Metema-Humera lowlands on black cracking soil were described as *Acacia seyal* - *Balanites aegyptiaca* forest and *Argeissus* - *Combretum* savannah woodlands [26], which have been transformed into big agricultural fields. During transformation of forest into fields, all trees except *Balanites aegyptiaca* were cleared in order to prevent gully erosion. Thus, agricultural fields in these areas have this tree species at about 25 m intervals (Figure 1). The soil in the study sites was black soil (eutric vertisols) [27] also reported to be associated with *P. orientalis* and kala-azar in Ethiopia and adjacent Eritrea and Sudan [2,12,27-29]. It is thought that such soil is hygroscopic and swells during the rainy season, then loses water and shrinks quickly during the dry season resulting in very deep cracks, creating essential microhabitats for the vector, *P. orientalis* [27,30]. Vertisol at shallow depths (45 cm) near *Balanites aegyptiaca* with relatively cool temperature, stable humidity and enhanced soil decomposition rate, were reported as suitable breeding site for sand fly larvae compared to open fields with seasonal 5–6°C temperature and 38.07% (average) relative humidity fluctuations [30]. The annual mean maximum temperature in Baeker and Humera towns vary from 29.10 to 41.2°C while the monthly mean minimum range from 13.50 to 25.40°C with November to May being the dry season that is characterized by high mean maximum temperatures (35.7 – 40.83°C), lack of heavy rain and cracking of black soil. March, April and May were the hottest months with mean maximum temperatures ranging from 38.9 to 40.83°C. The rainy season started in May and extended up to October with the highest rainfall occurred in August (Mean ± SD: 199.97 ± 126.53). Average annual rainfall received by the area from 2011 to 2013 was 791 mm [31].

### Nocturnal activities of *Phlebotomus orientalis*

A total of 22 CDC light traps were deployed on agricultural fields (n = 11), animal shelters (n = 3) and thickets of sparse *A. seyal* (n = 8), during January – May, 2013, to collect sand flies at one hourly intervals by changing sand fly cages before they were kept in separate tubes containing 96% ethanol for subsequent species identification. After *Sergentomyia* spp. were separated, *Phlebotomus* spp. were identified by using appropriate keys [32-34] following dissection and mounting of the specimens using Hoyer’s medium.

### Host preference of *Phlebotomus orientalis* using small rodents in Turner and Hoogstraal’s box traps

In order to evaluate the host preference of *P. orientalis* towards rodents, locally made box traps based on the design of Turner & Hoogstraal [35] were used. A total of 30 rodents (2 *Rattus* spp., 18 *Arvicanthis* spp., 4 *Tatera* spp., and 6 *Acomys* spp.) in 30 box traps were used as baits in Site 1 and 2 agriculture fields ( n = 10), animal shelter (n = 10) and thickets of *A. seyal* (n = 10) for a total of 10 days in March and April, 2013.

### Blood meal analysis of *Phlebotomus orientalis* using Reverse Line Blot (RLB) of cytochrome *b* PCR product

#### Sand fly collection and preservation

Any freshly blood-fed female *P. orientalis* encountered during previous sand fly collection from thickets of *A. seyal* (March, 2011) and dense mixed forest (July, 2011) in Baeker, was preserved for molecular identification of animal species that served as source of blood for this vector using Reverse Line Blot (RLB) of cytochrome *b* PCR product. For this purpose, the head of each fed female was mounted on the slides by Hoyer’s medium for later species identification. The remaining body (thorax and abdomen) was individually placed in empty sterile eppendorf tube with silica gel grains and cotton pads inside, bearing a corresponding label with the mounted specimen on the slide. They were stored at −20°C until molecular blood meal analysis was conducted in August 2012 in the Department of Microbiology & Molecular Genetics, Hadassah Medical School, Hebrew University, Israel.

#### DNA extraction

The thorax and abdomen of freshly-fed *P. orientalis* from each tube with silica gel were placed on filter and were transferred to test tube for digestion of tissues using lysis buffer (200 μl) and proteinase K (10 μl). Each *Phlebotomus orientalis* was homogenized using sterile wooden sticks and incubated in water bath (65°C) for 2 hours. Phenol extraction of DNA was made by adding 180 μl phenol before centrifugation at maximum speed (1,400 r.p.m) for 2.5 minutes. For ethanol DNA extraction, the aqueous part (150 μl) was transferred into a test tube containing sodium chloride solution (8 μl) before 400 μl cold (−20°C) ethanol was added and placed in refrigerator (−20°C) for 2 hours and cold centrifugation at maximum speed (1400 r.p.m) for 10 minute that precipitated DNA. Finally, pure DNA was obtained by removing the ethanol and suspending in 50 μl double distilled water.

#### Species-specific probes

This study described a blood meal identification approach based on PCR amplification of the mitochondrial cytochrome *b* gene (cyt *b*) followed by RLB analysis as already described [36,37]. RLB is a highly reproducible technique in which species-specific oligonucleotides (probes) are covalently linked to nylon membranes through the formation of amide bonds between the carboxyl group present on the membrane and amino-linked oligonucleotides which hybridizes with Biotinylated PCR products of the mitochondrial cyt *b* gene to reveal color that will be detected by colorimetric or enhanced chemiluminescent (ECL) systems. The species-specific oligonucleotide probes designed by Abassi et al. [36] for human (ATG CAC TAC TCA CCA GAC GC), cattle (ATT ATG GGT CTT ACA CTT T), sheep (TCC TAT TTG CGA CAA TAG CTT CCT), goat (ATA CAT ATC GGA CGA GGT CTA), camel (CGT TGG AAT TGT TTT ATT), donkey (CTA CTT TTC ACA GTT TAG CTA CA), dog (CAG ATT CTA ACA GGT TTA ), mouse (TGG AGT ACT TCT ACT GTT CGC AGT), rat (CAG TCA CCC ACA TCT GC), chicken (CAT CCG GAA TCT CCA C) and avian (TAC ACA GCA GAC AC) were used which adsorbed on the 3–4 mm strips of membrane in the following order from the top to bottom: Human, donkey, cattle, sheep, goat, camel, dog, mouse, rat, chicken and avian. One probe was found effective in identification of any avian species (36).

#### Polymerase chain reaction

To make 25 μl of final volume for PCR reaction, 20 μl master mix (1 μl forward primer, 1 μl backward primer and 18 ddH_2_O) and 5 μl DNA sample were mixed. PCR amplification of the mitochondrial cytochrome *b* gene was made using primers Cyto1 (5'-CCA TCA AAC ATC TCA GCA TGA TGA AA-3') and Cyto2 (5'- CCC CTC AGA ATG ATA TTT GTC CTC-3') before gel electrophoresis at 120 V in 1× TAE buffer in 1.5% agarose gels. The DNA fragments were visualized by UV light for determination of the sizes. The PCR product left from gel electrophoresis (18 μl) was used for RLB hybridization.

#### RLB hybridization

Species-specific probes from the 11 species were bounded on 5.5 × 15 cm biodyne C membrane (Gelman, USA). The species-specific probes were adsorbed on the membrane so that they would identify the corresponding PCR product indicating the species of animals *P. orientalis* fed. The membrane was first incubated with 0.1 M HCl and 10% EDAC solution before the bounding of probes and was later cut into strips of 3–4 mm width for the process of sample hybridization.

#### Statistical analysis

The nocturnal activities of *P. oreintalis* during 12 hours of the night were estimated from mean number *P. orientalis*/hour/CDC. All statistical analyses were performed using the Statistical Package of Social Science (SPSS), version 16. Comparisons of nocturnal activities during the night hours were analyzed using one-way analysis of variance (ANOVA). P-values less than 0.05 for mean number *P. orientalis*/hour/CDC were considered as significantly different.

## Results

### Population dynamics of *Phlebotomus orientalis*

**Figure 2:**
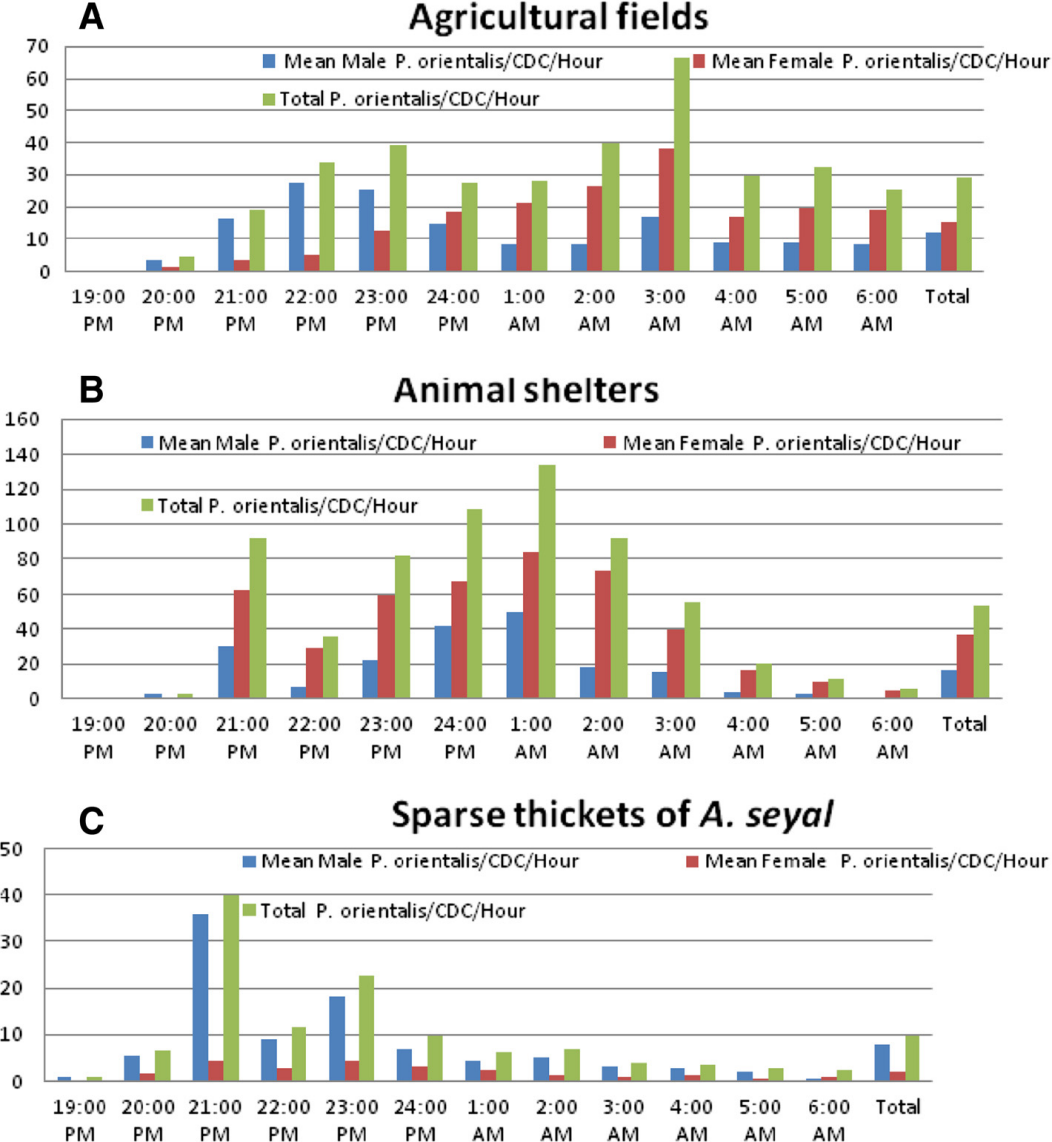
**Mean number of** ***Phlebotomud orientalis*** **/hour/night collected using CDC traps from agricultural fields (A), animal shelters (B) and thickets of** ***A. seyal*** **(C).**

A total of 6,083 *P. orientalis* (3,381 females and 2,702 males) was collected at one hour interval for 18 nights using a total of 22 CDC traps from agricultural fields near the camps (1,989 females and 1,614 males; n = 11 CDC traps), sparse thickets of *A. seyal* (167 females and 738 males; n = 8 CDC traps) and near sheep and goat shelters (1,349 females and 573 males; n = 3 CDC traps) during the study period. *Phlebotomus papatasi* (18), *P. duboscqi* (9), *P. begeroti* (7) and *P. rodhaini* (6) were other species of *Phlebotomus* collected with the same traps. Overall 20,236 (10,211 males and 10,025 females) *Sergentomyia* spp. were collected. The highest overall mean number of *P. orientalis*/hour/CDC trap was collected from animal shelters (53.37 ± 45.80) and followed by agricultural fields (28.87 ± 23.45). The lowest mean number of *P. orientalis*/hour/CDC trap was found from thickets of *A. seyal* (9.60 ± 16.38) (Figure 2). Nocturnal activities of female *P. oriental* were significantly different among the hours of the night in the agricultural fields (p = 0.023) and animal shelters (p = 0.001). However, no significant difference was found for the activities of *P. orientalis* at the different hours of the night in the thickets of *Acacia seyal* (p = 0.144). The peak activities of *P. orientalis* were at 1.00 a.m. near animal shelters (134.0 ± 7.21), 3.00 a.m. (66.33 ± 46.40) in the agricultural fields and 21.00 p.m. (40.06 ± 30.06) in thickets of *A. seyal.* The overall sex ratios of *P. orientalis* in agricultural field (1.30 female: 1 male), animal shelter (2.35 female: 1 male) and thickets of *A. seyal* (1 female: 4.42 male) were different. Thus, *P. orientalis* collections were male biased in thickets of *A. seyal* and female biased in around the camps.

### Host preference of *Phlebotomus orientalis* towards rodents using Turner and Hoogstraal’s traps

No *P. orientalis* was collected in rodent baited Turner Box’s trap. Only 5 *Sergentomyia* species (*S. schwetzi*) were collected from 30 Turner’s box trap deployed for 10 days in the agricultural fields, animal shelters and thickets of *A. seyal* in both sites.

### Host preference study of *Phlebotomus orientalis* based on molecular analysis of blood meals

**Figure 3:**
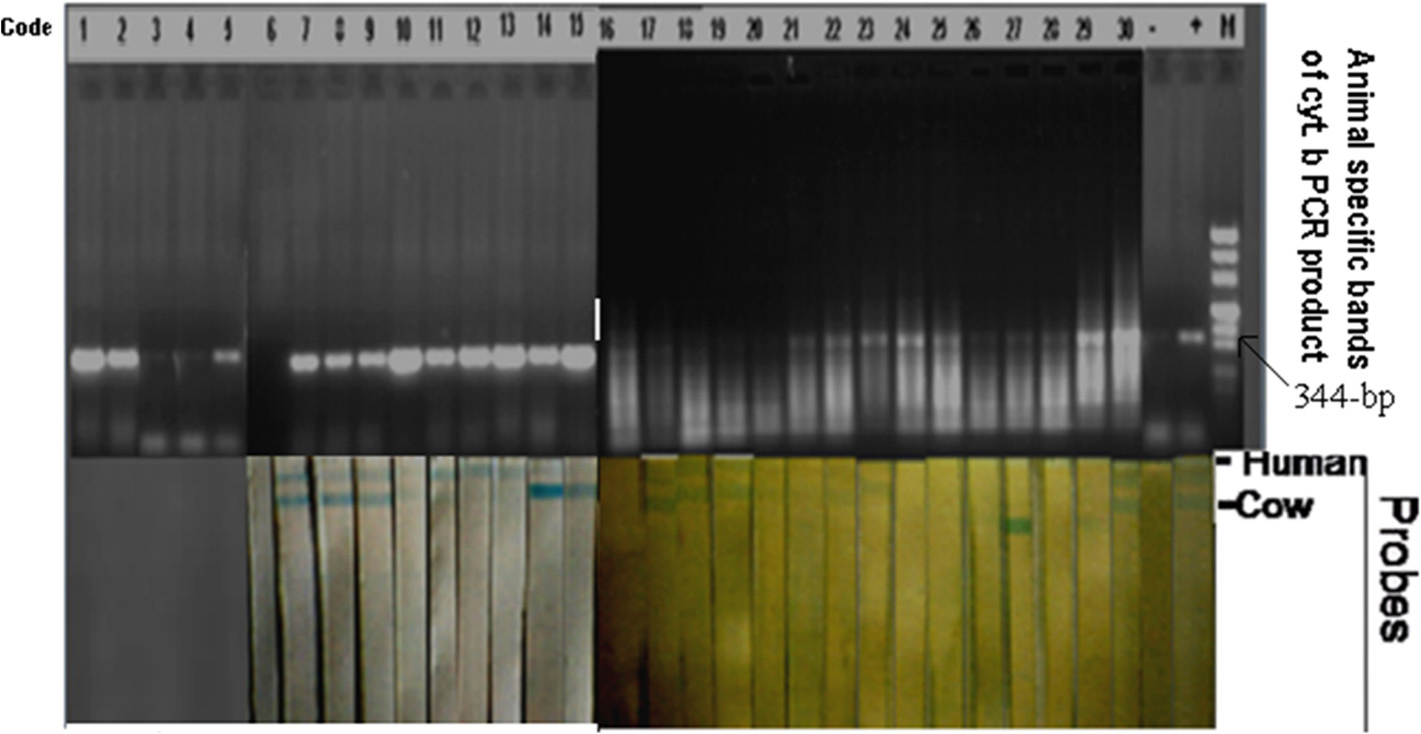
**Animal’s specific bands of cytochrome b PCR products (344-bp) obtained from blood meals of** ***Phlebotomus orientalis*** **on gel-electrophoresis after visualized by UV light (Top) and species specific hybridization of the PCR products obtained from different fresh fed** ***P. orientalis*** **using the different probes on nitrocellulose (bottom).** Only Human and cattle were found as source of blood meal for *P. orientalis* collected from *Acacia seyal* (March 2011) and dense mixed forest (July 2011) in the beaker site 1.

Only humans and cattle were found as the source of blood meals for *P. orientalis* collected from *A. seyal* and dense mixed forest in Baeker Site 1 from March to July 2011 when hybridization of PCR products of cytochrome *b* and the species specific probes was carried out. Of the 25 blood-fed *P. orientalis* analyzed using cyt *b* PCR and RLB (Figure 3), 7 (28%) were human, 9 mixed human and cattle (36%) and 2 cattle (8%). There were 7 samples (28%) with bands on agarose gel but not in nitrocellulose membrane indicating the existence of other animals outside the 11 probes.

## Discussion

So far no effective VL control measures have been conducted in Sudan and northwest Ethiopia, mainly due to the sylvatic nature of the habitats of adult *P. orientalis* and the lack of enough knowledge on transmission dynamics of *L. donovani* between unknown reservoir hosts and humans [38]. Studies on nocturnal and feeding activities of adult *P. orientalis* could give an insight about the behavior of this vector which is an essential component to design kala-azar control strategies. The results on nocturnal activities from hourly CDC and molecular blood meal analysis showed a risk of *P. orientalis* bite throughout the night as already reported [11]. Use of bed net by labour migrants involved in agricultural activities in the study areas is required.

The fact that the highest mean value of *P. orientalis* collected near animal shelters might indicate that goats and sheep were also sources of blood meals. The role of goats and sheep in the epidemiology of kala-azar is a subject of further study. Only one serological study in eastern Sudan indicated 8.5% *L. donovani* infection rate in goats using DAT [22]. In Kenya, *P. duboscqi* and *P. martini* were reported to be attracted to goats [39]. Recently, RLB of cytochrome *b* PCR products of blood meal collected from *P. orientalis* in domestic areas in Kafta-Humera district showed goats and sheep as a source of blood meal for this vector in addition to cattle (Yared et al., personal communication). The mean landing rate on human bait near goat and sheep shelters was almost twice the rates in the agricultural fields. Thus, goats and sheep might have zooprophylactic effect in preventing humans from kala-azar as, e.g. cattle [21]. This study has also shown that cattle to be important source of blood for *P. orientalis* in extra-domestic habitats where 72% of this species was found to have human and/or cattle blood-meal source. Mixed (human and cattle) blood meal was found to be very high (36%) which might indicate that *P. orientalis* had interrupted feeding on both hosts. Interruptions might have occurred due to inherent behavior of the female fly or due to the physical disturbance by the human or cattle hosts during the probing of the fly. Previous study has shown that cattle serve as the main source of blood for *P. orientalis* in domestic areas [23]. The reason why more cattle (92%) served as source of blood than human (2.2%) in domestic and predomestic areas using Enzyme Linked Immuno Sorbent Assay (ELISA) [23] in the area of Metema compared with this study is difficult to explain, but might need further investigation.

The bands of DNA fragments (cyt. *b*) on agarose gel (28%), that were not captured by the 11 probes used, may represent wild animals in the area. The probes that were not used include wild rodents and small carnivores. The existences of small carnivores such as genet cats and mongoose were confirmed during our study on reservoirs in these areas especially in the dense mixed forest (unpublished data). Either rodents or small carnivores or both could be the unknown source of blood for *P. orientalis*. Attractiveness towards mongoose (63.9 ± 12.1/CDC/night), genet cat (17.4 ± 3.72/CDC/night) and Nile rat (2.6 ± 0.56/CDC/night) compared to the control (0.4 ± 0.16/CDC/night) were reported in eastern Sudan [13]. No *P. orientalis* or other sandfllies were attracted to the rodents from all Turner and Hoogstraal box traps in this study. Similar study using CDC or sticky traps is required before reaching a conclusion.

The results of this study were in complete agreement with other observations [11,40] showing night-long human bite of *P. orientalis.* The nocturnal activities and human biting behavior was described as wave-like to show non-uniformity of biting rate at different hours of the night [11]. Such wave-like biting was due to the effect of wind. There were different trends of winds according to the observations during this study. Sometimes it began at 19:00 pm and ended at 21:00 p.m. or started at 21:00 p.m. and came down at 23:00 or 24:00 p.m. In few instances strong wind persisted until 24:00 p.m. Having understood this, long study period was used as the probability of unsuccessful sand fly collection was high.

## Conclusion

*Phlebotomus orientalis* could bite humans at different hours of the night and there is a risk of kala-azar infection in agricultural fields, thickets of *A. seyal* and in the camps. It is also attracted to domestic animals in the extra-domestic habitats for blood meal. *Phlebotomus orientalis* is most probably catholic in its feeding habits and having wide range of mammals as blood meal sources depending on the available host in space and time. Whether these animals are potential carriers of *Leishmania* causing Kala-azar needs further investigation.

### Recommendations

Despite several attempts made to investigate the reservoir host of *L. donovani* in Sudan and northwest Ethiopia, further study, with more emphasis on wild animals in extra-domestic animals, are required.
